# Osteosarcoma of the patella: A case report

**DOI:** 10.3109/03009734.2011.649865

**Published:** 2012-08

**Authors:** Shuichi Chida, Hiroyuki Nagasawa, Kyoji Okada, Yoichi Shimada

**Affiliations:** ^1^Department of Orthopedic Surgery, Akita University Graduate School of Medicine, 1-1-1 Hondo, Akita, 010-8543, Japan; ^2^Department of Physical Therapy, Akita University Graduate School of Medicine, 1-1-1 Hondo, Akita, 010-8543, Japan

**Keywords:** Osteosarcoma, patella, treatment

## Abstract

We present a rare case of osteosarcoma involving the patella. A 30-year-old Japanese woman first consulted our out-patient clinic with a 2-year history of knee pain. Radiographs showed an enlargement of the patella with irregular distribution of both osteolytic and sclerotic lesions. Computed tomography and magnetic resonance imaging demonstrated soft tissue extension at the anterior part of the patella. Incisional biopsy showed abundant osteoid formation by spindle-shaped malignant cells, and the histological diagnosis was conventional osteosarcoma. The patient underwent preoperative chemotherapy, but there was no response. Furthermore, she developed a pathological fracture during chemotherapy. She underwent above-the-knee amputation with postoperative chemotherapy. She developed multiple metastases in the thoracic vertebrae 20 months after the surgery. At the most recent examination, she remains alive with multiple spinal metastases without paralysis 4 years after the surgery.

## Introduction

The patella is an uncommon site for primary bone tumor, and benign tumors are more common than malignant tumors (1). Osteosarcoma of the patella is extremely rare, and six such cases have been reported. We report a case of osteosarcoma of the patella with a review of the literature.

## Case report

In June 2004, a 30-year-old Japanese female initially consulted a local physician with a 6-month history of right knee pain and was treated with a non-steroidal anti-inflammatory drug. In December 2005, she consulted another local clinic since the knee pain had increased and was referred to our out-patient clinic.

**Figure 1.**
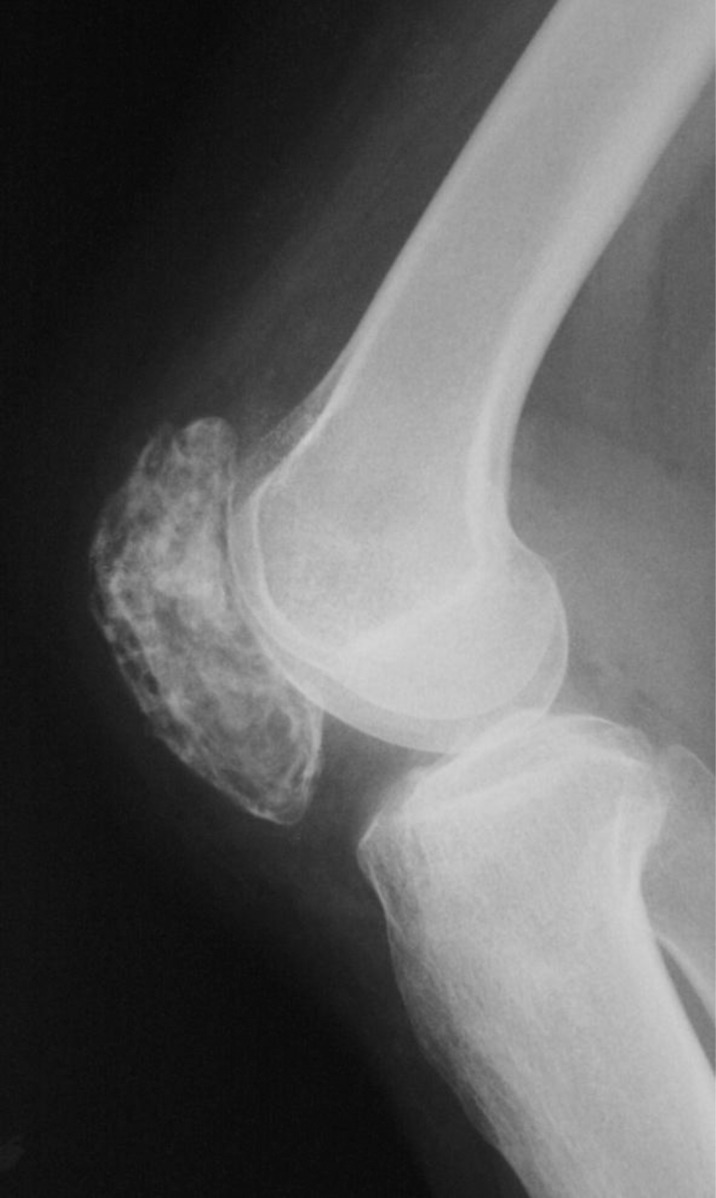
A lateral radiograph of the right knee. The right patella was enlarged, and its cortical shell was irregularly discontinued. Osteolytic and osteoblastic lesions were irregularly distributed in the patella.

**Figure 2.**
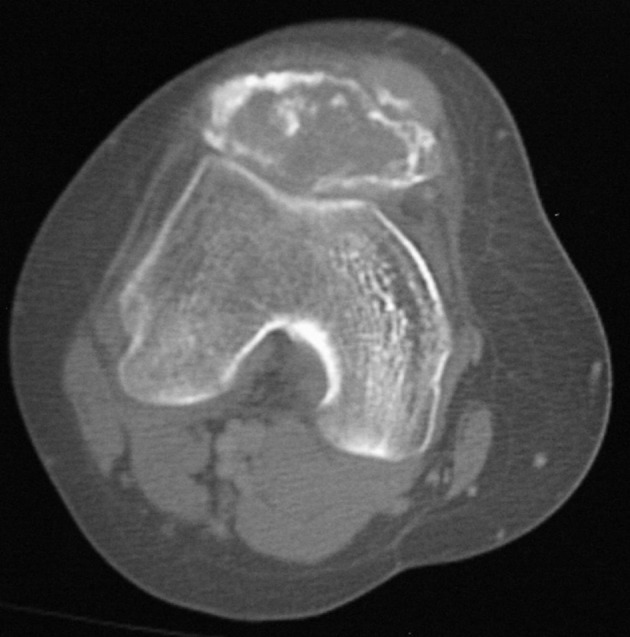
Computed tomography showing an intraosseous osteolytic lesion and soft tissue extension at the anterior part to the patella.

**Figure 3.**
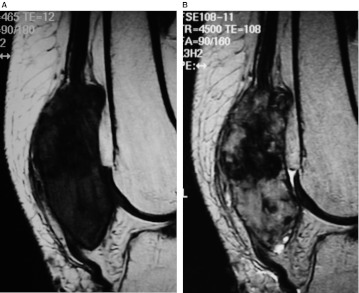
Magnetic resonance imagines showing an intraosseous lesion extending into the anterior soft tissue. A: On T1-weighted images, the lesion showed low signal intensity. B: On T2-weighted images, the proximal part of the lesion showed low signal intensity with partial high-signal areas, and the distal part showed high signal intensity with focal low-signal areas.

**Figure 4.**
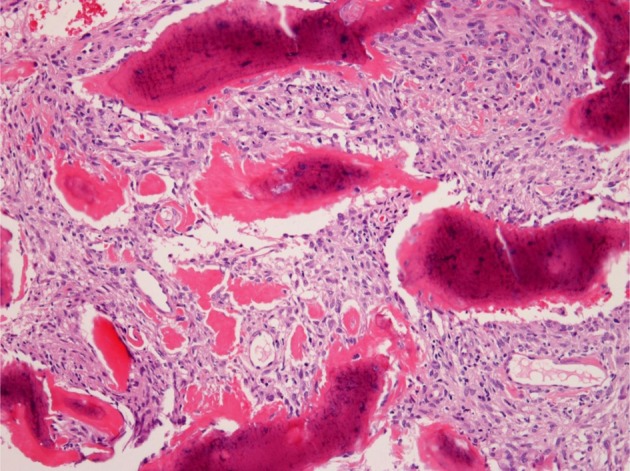
Middle-power view of the biopsied specimens of the patella showing a proliferation of spindle-shaped atypical cells with condensation of chromatin and abundant osteoid formation (×100, hematoxylin and eosin).

On physical examinations, redness and swelling of the anterior aspect of the knee were noted, and flexion of the knee joint was restricted to 120°. She could not raise her lower leg against gravity because of muscle atrophy around the right thigh. On radiographs, the right patella was enlarged, and the cortical shell was irregularly discontinuous. Osteolytic and osteoblastic lesions were irregularly distributed in the patella (Figure 1). Computed tomography showed an intraosseous osteolytic lesion and soft tissue extension at the anterior part to the patella (Figure 2). Magnetic resonance imagings also demonstrated an intraosseous lesion extending into the anterior subcutaneous tissue. The lesion showed low signal intensity on T1-weighted images. On T2-weighted images, the proximal part of the lesion showed low signal intensity with partial high-signal areas, and the distal part showed high signal intensity with focal low-signal areas (Figure 3). Incisional biopsy demonstrated a proliferation of spindle-shaped atypical cells with condensation of chromatin and abundant osteoid formation (Figure 4). The histological diagnosis was fibroblastic osteosarcoma.

The patient underwent chemotherapy including high-dose ifosfamide, adriamycin, and cisplatin. However, these were not clinically effective, and the response was estimated as progressive disease. Furthermore, she developed a pathological fracture of the patella during chemotherapy. Therefore, we performed above-the-knee amputation in April 2006. The surgical specimen showed extensive remnant of tumor cells, and the effect of the chemotherapy was evaluated as ‘poor’. High-dose methotrexate, adriamycin, and cisplatin were administrated postoperatively. In December 2007, she developed multiple metastases in the thoracic vertebrae. She again underwent chemotherapy including high-dose ifosfamide, adriamycin, and cisplatin. At the most recent examination in 2010, she remains alive with metastatic disease in the thoracic spine without paralysis.

## Discussion

Among primary malignant bone tumors, osteosarcoma is the most frequent. Osteoid and/or bone produced by malignant cells is fundamental to making a diagnosis of osteosarcoma. The metaphyseal parts of the long bone, especially the region of the knee, are common sites. Although osteosarcoma shows a predilection for both the distal femur and proximal tibia, the patella is not a common site. In the Mayo Clinic series, there was only one case showing an osteosarcoma of the patella among 1649 cases of osteosarcoma (2). Benign tumors are more frequent than malignant tumors in patella (73% versus 27%) (1). The patella is considered to have similarity in ossification with the epiphysis of the long bone. It may be reasonable that giant cell tumors and chondroblastomas, which are regarded as epiphyseal tumors, are the most common in patella (1,3–5). Among malignant neoplasms of the patella, hemangioendothelioma, lymphoma, metastasis, and osteosarcoma were reported (2).

To our knowledge, eight cases of osteosarcoma in the patella, including the current case, have been reported in the English literature (Table I). Characteristically, three of the eight cases were associated with factors linked to the development of osteosarcoma, such as post-radiation status (case 3), Rothmund–Thomson syndrome (case 4), and Werner syndrome (case 5), respectively (6–8). Furthermore, in contrast to conventional osteosarcomas, the ages of the eight patients were relatively high. Patient ages at diagnosis ranged from 18 years to 54 years (median, 38 years), and five of the eight patients (75%) were in the fourth (cases 2, 5, and 8) or sixth (cases 3, 6, and 7) decade of life. This age distribution may be caused by pre-existing conditions such as irradiation and genetic disorder.

**Table I.** Details of eight cases on osteosarcoma of the patella.

Author, year (ref)	Age/Sex	Interval^a^	M0/1	Initial Tx	Biopsy	Second Tx	Associated factors	Prognosis
1. Goodwin, 1961 (11)	24/ M	6 months	0	Patellectomy	–	AKA	None	DOD
2. Nagai, 1993 (9)	34/F	7 months	M1	Steroid injection	+	Chemo + AKA	None	1 month, DOD
3. Okada, 1994 (6)	54/M	3 months	0	Chemo + patellectomy	+		Post-radiation	120 months, NED^a^
4. Ferguson, 1997 (7)	18/F	NA	0	Patellectomy	–	Knee resection	Rothmund–Thomson syndrome	25 months, NED
5. Ishikawa, 2000 (8)	35/F	NA	NA	NA	NA	NA	Werner syndrome	NA
6. McGrath, 2006 (10)	53/M	3 months	M1	Steroid injection	+	Chemo + Knee resection	None	15 months, DOD
7. Cho, 2009 (12)	53/F	3 years	0	NA	+	Reconstruction after patellectomy	None	26 months, NED
8. The current case	31/F	2 years	0	NSAIDs	+	Chemo + AKA	None	48 months, AWD

^a^Interval from the onset of symptoms to definitive diagnosis.

^b^Personal communication from the authors. AKA = above-the-knee amputation; AWD = alive with disease; Chemo = systemic chemotherapy; DOD = died of disease; NA = not available; NED = no evidence of disease; NSAIDs = non-steroidal anti-inflammatory drugs; Tx = treatment.

Of the eight cases, clinical courses were available in seven. Six of the seven patients presented with pain and swelling of the patella, whereas the current case initially presented with anterior knee pain only. The interval from onset of symptoms to definitive diagnosis ranged from 3 months to 3 years (median 13 months) and tends to be prolonged due to misdiagnosis or failure to consider the potential for malignancy. In only one patient (case 3) was biopsy performed at the initial presentation. The patient subsequently underwent chemotherapy and surgery and has remained well for 120 months to date (Okada et al., with recent personal communication) (6). Two cases (cases 2 and 6) that had metastatic lesions at the initial presentation were managed in the same manner as case 3 (9,10). However, both patients died of lung metastasis. In the remaining three cases, including the current case, a biopsy was not initially performed, and these patients initially underwent inadequate treatment such as patellectomy with inadequate surgical margin (cases 1 and 4) (7,11) or conservative management (current case). Of these three cases, one (case 1) died of metastatic disease, one (current case) remains alive with metastatic disease, and one (case 4) has remained alive and well for 24 months to date. Delay in the diagnosis and inadequate management at the initial presentation might be related to the poor prognosis.

Ferguson et al. recommended that a definitive diagnosis should always be obtained before any treatment of the bone lesion is initiated (7). Case 3, who was diagnosed by biopsy prior to the start of any treatment and was treated with both preoperative and postoperative chemotherapy, has shown a good clinical result. The importance of a definitive diagnosis by a biopsy should be stressed when encountering patella tumors in clinical practice.
